# The Long Term Impact of Cataract Surgery on Quality of Life, Activities and Poverty: Results from a Six Year Longitudinal Study in Bangladesh and the Philippines

**DOI:** 10.1371/journal.pone.0094140

**Published:** 2014-04-18

**Authors:** Lisa Danquah, Hannah Kuper, Cristina Eusebio, Mamunur Akm Rashid, Liza Bowen, Allen Foster, Sarah Polack

**Affiliations:** 1 Faculty of Infectious and Tropical Diseases, London School of Hygiene & Tropical Medicine, London, United Kingdom; 2 Cataract Foundation of the Philippines, Bacolod, Philippines; 3 Dhaka Community Medical College, Dhaka, Bangladesh; Zhongshan Ophthalmic Center, China

## Abstract

**Background:**

Cataract surgery has been shown to improve quality of life and household economy in the short term. However, it is unclear whether these benefits are sustained over time. This study aims to assess the six year impact of cataract surgery on health related quality of life (HRQoL), daily activities and economic poverty in Bangladesh and The Philippines.

**Methods and Findings:**

This was a longitudinal study. At baseline people aged ≥50 years with visual impairment due to cataract (‘cases’) and age-, sex-matched controls without visual impairment were interviewed about vision specific and generic HRQoL, daily activities and economic indicators (household per capita expenditure, assets and self-rated wealth). Cases were offered free or subsidised cataract surgery. Cases and controls were re-interviewed approximately one and six years later. At baseline across the two countries there were 455 cases and 443 controls. Fifty percent of cases attended for surgery. Response rates at six years were 47% for operated cases and 53% for controls. At baseline cases had poorer health and vision related QoL, were less likely to undertake productive activities, more likely to receive assistance with activities and were poorer compared to controls (p<0.05). One year after surgery there were significant increases in HRQoL, participation and time spent in productive activities and per capita expenditure and reduction in assistance with activities so that the operated cases were similar to controls. These increases were still evident after six years with the exception that time spent on productive activities decreased among both cases and controls.

**Conclusion:**

Cataract causing visual loss is associated with reduced HRQoL and economic poverty among older adults in low-income countries. Cataract surgery improves the HRQoL of the individual and economy of the household. The findings of this study suggest these benefits are sustained in the long term.

## Introduction

Age related cataract is the leading cause of blindness in low-income settings [1]. The aim of cataract surgery is to improve the vision and thereby the quality of the daily lives of people affected. However, relatively little is known about the extent to which this occurs in the long term in low income settings. Blindness and poverty are closely linked; people who are blind may experience restricted opportunities to engage in income generating activities [2], [3] and people who are poor are less likely to be able to afford sight restoring cataract surgery [4], [5], [6]. However, empirical data on whether sight restoration impacts on economic poverty in the long term is lacking.

The ‘Cataract Impact Study’ was a longitudinal intervention study undertaken to explore the impact of cataract surgery on health related quality of life (HRQoL), daily activities and economic poverty among adults aged ≥50 years in Kenya, the Philippines and Bangladesh [7], [8], [9]. People with visual impairment from cataract (‘cases’) and age- gender- matched peers without visual impairment from cataract (‘controls’) were identified through population based surveys [4], [5], [6] and interviewed about their HRQoL, daily activities and poverty indicators. All cases were offered free or subsidized surgery. Approximately one year later cases and controls were re-interviewed. At baseline, this study found that compared to people without visual impairment:

- people with cataract had poorer vision specific and generic health related quality of life [10], [11], [12]
- people with cataract were less likely to engage in and spent less time on productive activities and were more likely to have assistance with daily activities [2]
- people with cataract were poorer in terms of assets, self-rated wealth and household per capita expenditure [13]

One year after cataract surgery this study found that compared to baseline:

- the vision-specific and generic health-related quality of life of people with cataract who had undergone cataract surgery had improved [8]
- people with operated cataract were more likely to undertake and spent more time on productive activities and were less likely to report receiving assistance with activities [7]
- the per capita expenditure of households with people with operated cataract had increased [9]

The cataract impact study highlighted some key benefits of cataract surgery to a population of older adults in low-income countries after one year. However, it is unclear whether these benefits are sustained over time, particularly considering the older age group mainly affected who may be more likely to experience co-morbidities. Few studies have explored the long term impact of cataract surgery on quality of life outcomes [14], [15] and no information on long-term impact on time-use or poverty indicators have been identified.

Three different indicators of poverty were included in the Cataract Impact Study: household expenditure, asset ownership and self-rated wealth. While increases in household expenditure were observed among operated cases at one year follow up, asset ownership and self-rated wealth remained largely unchanged. This is unsurprising since they are longer term measures of poverty; more time is needed for households to accumulate additional assets or for perceptions of wealth to change. A longer follow-up is therefore necessary to explore the full impact of cataract surgery on poverty.

In the current study, a six year follow up of the Cataract Impact Study was undertaken in Bangladesh and the Philippines to explore the long term impact of cataract surgery on HRQoL, activities and poverty.

## Methods

### Study setting

This study was conducted in one district in Bangladesh (Satkhira) and two areas of the Philippines (Negros Island and Antique district, Panay Islay). A third study site (Nakuru, Kenya) included in the original Cataract Impact Study was not included in the six year follow up because of the smaller study numbers recruited at baseline.

### Study population

Participants in the Cataract Impact Study were identified primarily through population-based blindness surveys which included >3600 people aged ≥50years in each setting [4], [6]. Clusters of 50 people aged ≥50years were selected by probability proportionate to size sampling and within clusters, households were selected using compact segment sampling [16]. Visual acuity was assessed using tumbling E-chart the examination for cataract was conducted by an ophthalmologist using a direct ophthalmoscope. People in the survey with pinhole corrected visual acuity (VA) <6/24 in the better eye due to cataract were eligible to be cases. For each case identified, one (or up to two people in Bangladesh) age and gender matched control without visual impairment was randomly selected from the same cluster. Due to logistical and time constraints, additional case finding was undertaken in the community to increase the number of cases included.

### Intervention

All cases were offered cataract surgery at the local hospital. In Bangladesh surgery was free and transport was provided by the hospital. In the Philippines a fee was requested, but those who could not afford the fee were offered free surgery. Patients made their own travel arrangements, but were reimbursed travel costs.

### Data collection

Baseline surveys were conducted during 2005–2006. The first follow up was conducted one year later and the current study was conducted in September (Philippines) and November (Bangladesh) 2011 during the same climatic seasons as the baseline. Interviews were conducted in participants' homes by trained interviewers in the local language.

#### Measures of Health Related Quality of Life

We measured a) vision related QoL and b) generic HRQoL. Vision related quality of life was measured using the WHO/PBD VF20. The scale includes 20 items on overall eyesight, general functioning and psychosocial well-being, each with a 5-point response option. Evidence for reliability and validity of this scale was found at baseline [10], [11], [12]. Generic HRQL was measured using the European Quality of Life (Euroqol) questionnaire which has shown to be valid and reliable in different settings [17], [18], [19]. The questionnaire includes two components [20]. The first (EQ-5D) consists of five domains (mobility, self-care, usual activity, pain/discomfort and anxiety/depression) each with three response options (no problem, some problem or extreme problem). The second component measures self-rated health using a visual analogue scale. Participants are asked to rate their ‘health today’ on a scale ranging from 0 (‘worst imaginable health state’) to 100 (‘best imaginable health state’). Both HRQoL tools were translated into the target languages in each country using standard forward and backward translation methods [10], [12]. Translations of the generic HRQoL tool were undertaken independently of the Euroqol group and have not therefore been approved by the Euroqol group.

#### Measures of activities

Activity data were collected using the ‘stylised activity list’ developed for the World Bank's Living Standards Measurement Survey [21]. Participants were asked whether they had been involved in each of a preset list of common daily activities during the last week and if they had, whether they had been involved in the activity yesterday. Those who had been involved in an activity ‘yesterday’ were asked to estimate how much time they had spent on the activity and whether they received any assistance from another person in performing that activity. Activities were grouped as follows:

Productive activities: household/family activities (cooking/washing dishes, cleaning house/clothes, shopping, looking after children/elderly/sick), paid work (paid employment, commission work, self-employed/own business); work for own use (agriculture, animal rearing, fetching firewood/water, processing agricultural products/food) other
Leisure outside the home: social visits, attending ceremonies, attending meetings
Leisure inside the home: reading/listening to radio/watching TV; chatting, relaxing with friends/family; prayer (Bangladesh), other
No activity

#### Measures of poverty

Three different poverty indicators were measured: a) household per capita expenditure (PCE), b) asset ownership and c)self-rated health. Interviews were conducted with the person primarily responsible for household finances.

- PCE was measured using methods based on the World Bank's Living Standards Measurement Survey [22]. The household informant was asked to recall the monetary value of food that was purchased, consumed from home production and received as payment in kind or as gifts by all household members. The time period for which this was measured was the past week for frequently consumed items (scaled up to estimate monthly consumption) and the past month for less frequently consumed items. In addition, data were collected about monthly expenditure on education, health, household and personal items and rent paid (or rental equivalent for home owners). In total 90 items were included in the questionnaire for the Philippines and 79 in Bangladesh. The consumption on all items was summed to calculate a monthly household total. PCE was calculated by dividing total consumption by the number of household members.
- Asset ownership: data were collected on the number and type of different context-specific assets owned by the household (e.g. cattle, furniture, electrical goods, vehicles) and household characteristics (e.g. building materials of the floor, roof and walls; type of toilet and number of rooms). These data were used to develop a relative index of household assets using principal components analysis (PCA) [23].
- Self-rated wealth: the household informant was asked to rank the household's wealth relative to others in the community from 1(poorest) to 10 (richest)

### Covariates

We collected standard socio-demographic variable data including age, gender, education, literacy and marital status.

### Ethical considerations

Informed signed or thumb-printed consent was obtained from all cases and controls. All cases with operable cataract (VA<6/24) were referred for surgery. Ethical approval was obtained from the ethics committees of the London School of Hygiene & Tropical Medicine, the Bangladesh Medical Research Council and the University of St. La Salle, Bacolod, Philippines. The use of thumb-printed consent for participants who were unable to write was approved by the ethics committees.

### Analysis

Data analyses were restricted to cases and controls included in all three data collection times (baseline, one year and six year follow up). Data on household expenditure were cleaned, excluding gross outliers and imputing rental equivalents based on household characteristics and non-rent expenditure where these estimates were missing or unreasonably low (<$1 per month – 34 in total across the two settings). PCE for the one and six year follow up surveys were deflated by the cumulative inflation rates over that time so that they were comparable to baseline. This adjusted PCE was converted into US dollars using the exchange rate at the time of the baseline survey. The mean proportion of time spent on each activity group was calculated by dividing total minutes on specific activity group by the sum of minutes reported on all activities for that individual, as not all totals were exactly 24 hours. To ease interpretation, the proportion of time was converted into hours and minutes.

We compared the baseline socio-demographic and clinical characteristics of a) cases and controls and b) cases and controls included and lost to follow up using t-tests for continuous variables and chi-squared for categorical variables. Change in outcomes over time were assessed using the paired t-test for continuous variables (log transformed PCE, assets, vision related QoL, self-rated health and time spent) and McNemars test for categorical variables (EQ-5D responses and participation in activities). We compared outcomes between cases and controls at each time point using firstly t-tests and chi square/fishers exact tests and secondly multivariate regression to adjust for age and gender. As the adjusted and unadjusted results were similar only the unadjusted findings will be presented here. PCE data were skewed and so were log transformed for analysis. Vision related QoL and data on amount of time spent on productive activities were also skewed, but could not be transformed, so analyses of these data were repeated using non-parametric tests (Wilcoxon sign rank and Mann-whitney U test). Findings were essentially unchanged and therefore parametric results are presented.

## Results

### Study population

The baseline survey included 217 cases and 280 controls in Bangladesh and 238 cases and 163 controls in the Philippines. Uptake of cataract surgery was generally low: 54% of cases (n = 117) identified at baseline attended for surgery by one year in Bangladesh and 47% (n = 112) in the Philippines. Only a small proportion (<5%) of the un-operated cases at one year had received cataract surgery at six years and they were excluded from the analysis. At the six year follow up the response rate in Bangladesh was 48% (n = 56) for operated cases and 51% (n = 142) for controls, and in the Philippines, 45% (n = 51) for operated cases, and 56% (n = 91) for controls. Of those lost to follow up 63% of cases and 56% of controls had died in Bangladesh (p = 0.32) and 75% of cases and 47% of controls had died in the Philippines (p = 0.001).

Operated cases (herein after referred to as ‘cases’) were similar to controls in terms of socio-demographic variables, with the exception that cases were more likely to be female (p = 0.02) in the Philippines and were less likely to have an education or be literate in Bangladesh (table 1).

**Table 1:** Baseline socio-demographic characteristics of cases and controls included in the one and six year follow-up.

	THE PHILIPPINES			BANGLADESH		
	Operated cases	Controls	P-value	Operated cases	Controls	P-value
	n = 51	n = 91		n = 56	n = 142	
**Mean age (years)**	69.1 (66.6–71.7)	68.3 (66.4–70.1)	0.58	68.5 (66.1–70.9)	66.7 (65.4–68.1)	0.18
**Female**	73%	53%	0.02	55%	59%	0.63
**Married**	49%	63%	0.12	50%	58%	0.28
**Education**	90%	92%	0.66	18%	39%	0.005
**Literate**	88%	89%	0.89	9%	30%	0.002
**Visual acuity**						
≥6/18	0%	100%	N/A	0%	100%	N/A
<6/24–6/60	24%			27%		
<6/60–3/60	20%			20%		
<3/60	56%			53%		

There were few differences in socio-demographic, visual acuity, HRQoL, activity and poverty variables between participants included and lost to follow up. The exceptions were that cases lost to follow up (Bangladesh only, p = 0.04) and controls (Bangladesh p = 0.04 and the Philippines p = 0.007) were slightly older than those who were traced, in the Philippines male cases were slightly more likely to be lost to follow up than females (p = 0.03) and in Bangladesh cases lost to follow up spent slightly less time on productive activities (p = 0.02) (data not presented).

### Health related Quality of Life

#### Vision related quality of life

At baseline cases had substantially poorer overall eyesight, general functioning and psychosocial scores compared to controls (p<0.001, table 2). At the one year follow up, all scores among cases increased significantly up to the level of controls and they remained significantly higher compared to baseline after six years (p<0.001). For example in the Philippines mean general functioning scores among cases increased from 27.1 at baseline to 86.1 at one year and was 79.6 at the six year follow up. Among controls scores were 88.3 at baseline, 85.9 at one year and 82.7 at six year follow up. Some reductions in vision related QoL scores were observed between one and six years for both cases and controls, but scores among cases remained substantially higher than at baseline and did not differ significantly from controls at either follow up.

**Table 2:** Vision related quality of life at baseline and one and six year follow up for operated cases and controls.

	THE PHILIPPINES			BANGLADESH		
	Operated cases	Controls		Operated cases	Controls	
	Means (95% CI)	Means (95% CI)	P-value: Cases versus controls	Means (95% CI)	Means (95% CI)	P-value: Cases versus controls
**Eyesight rating**						
Baseline	18.6 (12.9–24.4)	65.4 (60.1–70.7)	<0.001	12.5 (9.1–15.9)	64.6 (61.6–67.7)	<0.001
1 year follow up	75.5 (69.3–81.7)	75.5 (69.8–81.3)	0.99	75.0 (69.3–80.7)	74.0 (70.7–77.1)	0.74
6 year follow up	70.1 (62.4–77.8)	75.8 (71.0–80.6)	0.19	64.5 (58.5–70.6)	65.6 (61.7–69.5)	0.77
P-value: 1 year versus baseline	<0.001	0.003		<0.001	<0.001	
P-value: 6 year versus 1 year	0.17	0.93		<0.001	<0.001	
**General Functioning**						
Baseline	27.1 (21.0–33.3)	88.3 (85.4–91.1)	<0.001	16.8 (12.9–20.8)	86.3 (83.8–88.8)	<0.001
1 year follow up	86.1 (81.5–90.7)	85.9 (82.7–89.2)	0.95	82.0 (75.6–88.4)	82.1 (79.2–85.1)	0.95
6 year follow up	79.6 (72.3–87.0)	82.7 (78.7–86.8)	0.43	73.8 (66.8–80.8)	75.8 (71.9–79.7)	0.61
P-value: 1 year versus baseline	<0.001	0.08		<0.001	0.007	
P-value: 6 year versus 1 year	0.02	0.09		<0.001	0.002	
**Psychosocial**						
Baseline	40.4 (34.4–46.5)	87.4 (84.1–90.6)	<0.001	32.7 (26.0–39.4)	89.9 (87.4–92.4)	<0.001
1 year follow up	83.8 (79.6–88.1)	87.8 (84.3–91.4)	0.16	85.9 (79.8–92.0)	83.0 (79.8–86.2)	0.36
6 year follow up	75.9 (68.6–83.1)	78.1 (73.5–82.7)	0.58	69.9 (63.0–76.7)	69.9 (65.5–74.1)	0.99
P-value: 1 year versus baseline	<0.001	0.72		<0.001	<0.001	
P-value: 6 year versus 1 year	0.02	<0.001		<0.001	<0.001	

#### Generic health related quality of life

At baseline in both settings, cases were significantly more likely to report problems in each EQ-5D domain, with the exception of pain/discomfort in Bangladesh (table 3). At the one year follow up the proportion of cases reporting problems reduced significantly by at least 20% in 4 of the 5 domains in the Philippines (mobility, self-care, daily activities, pain/discomfort) and 3 of the 5 domains in Bangladesh (mobility, self-care, daily activities) compared to baseline. These reductions in reported problems among cases remained six years later.

**Table 3:** Proportion of respondents reporting some (any/extreme) problem with EQ-5D domains at baseline and one and six year follow up for operated cases and controls.

	THE PHILIPPINES			BANGLADESH		
	Operated cases	Controls		Operated cases	Controls	
	n = 51	n = 91		n = 56	n = 142	
EQ-5D Domain	%	%	P-value: Cases versus controls	%	%	P-value: Cases versus controls
**Mobility**						
Baseline	82%	57%	0.002	88%	43%	<0.001
1 year follow up	59%	52%	0.41	34%	44%	0.18
6 year follow up	55%	46%	0.32	44%	36%	0.36
P-value: 1 year versus baseline	0.01	0.38		<0.001	<0.001	
P-value: 6 year versus 1 year	0.81	0.38		0.23	0.11	
**Self-care**						
Baseline	57%	10%	<0.001	66%	18%	<0.001
1 year follow up	31%	23%	0.28	26%	23%	0.60
6 year follow up	41%	34%	0.40	**27%**	**27%**	0.98
P-value: 1 year versus baseline	0.01	0.005		<0.001	0.29	
P-value: 6 year versus 1 year	0.19	0.07		0.78	0.41	
**Daily activities**						
Baseline	39 (76%)	26 (29%)	<0.001	50 (89%)	60 (42%)	<0.001
1 year follow up	23 (45%)	26 (29%)	0.05	24 (43%)	53 (37%)	0.47
6 year follow up	28 (55%)	37 (41%)	0.10	23 (42%)	46 (33%)	0.24
P-value: 1 year versus baseline	0.01	1.0		<0.001	0.32	
P-value: 6 year versus 1 year	0.28	0.05		0.99	0.40	
**Pain/discomfort**						
Baseline	49 (96%)	60 (65%)	<0.001	44 (79%)	120 (85%)	0.32
1 year follow up	38 (75%)	65 (71%)	0.69	44 (78%)	117 (82%)	0.53
6 year follow up	38 (75%)	59 (65%)	0.24	49 (89%)	120 (86%)	0.53
P-value: 1 year versus baseline	0.005	0.37		0.99	0.59	
P-value: 6 year versus 1 year	1.0	0.24		0.08	0.40	
**Anxiety/depression**						
Baseline	71%	52%	0.03	91%	78%	0.03
1 year follow up	61%	41%	0.02	86%	89%	0.46
6 year follow up	37%	44%	0.42	91%	91%	0.91
P-value: 1 year versus baseline	0.25	0.12		0.32	0.01	
P-value: 6 year versus 1 year	<0.001	0.34		0.99	0.004	
Baseline	0.003	0.62		0.32	0.51	

Cases had significantly poorer baseline self-rated health scores compared to controls (Philippines: cases 53.9 vs controls 63.1, p = 0.001; Bangladesh 51.8 vs 59.7, p = 0.02). Self-rated health among cases increased at the one year follow up (Philippines 57.9; Bangladesh 70.0) compared to baseline so that they were no longer significantly different to controls. The increase in self-rated health was sustained after six years in Philippines (59.2). In Bangladesh scores decreased among both operated cases (55.7) and controls (50.7) between one and six years, but scores for cases remained significantly higher compared to baseline.

### Activities

#### Participation in activities during the previous week

Nearly all cases and controls (>95%) participated in leisure activities inside the home and ‘inactivity’ and therefore these categories were not included in the analyses. At baseline cases were significantly less likely to take part in productive activities compared to controls in both settings (80% cases, 97% controls in Philippines, p = 0.002; 61% cases and 97% controls in Bangladesh p = <0.001, Table 4). At both one and six year follow ups the proportion of cases engaged in productive activities was significantly higher compared to baseline (by 14% and 27% at one year and 8% and 18% at six years in Philippines and Bangladesh respectively) so that there were no significant differences between cases and controls at either follow up. For leisure outside of the house, participation was similar for cases and controls at each time point.

**Table 4:** Participation in different activities over the past week at baseline and one and six year follow up for operated cases and controls.

	THE PHILIPPINES			BANGLADESH		
	Operated cases	Controls		Operated cases	Controls	
	n = 51	n = 91		n = 56	n = 142	
	%	%	P-value: Cases versus controls	%	%	P-value: Cases versus controls
**Productive activities**	%					
Baseline	80%	97%	0.002	61%	97%	<0.001
1 year follow up	94%	96%	0.70	88%	94%	0.13
6 year follow up	88%	88%	0.96	79%	83%	0.46
P-value: 1 year versus baseline	0.02	0.65		0.001	0.15	
P-value: 6 year versus 1 year	0.18	0.02		0.09	0.001	
**Leisure outside house**						
Baseline	47%	45%	0.82	43%	40%	0.73
1 year follow up	67%	77%	0.18	70%	70%	0.99
6 year follow up	65%	70%	0.49	45%	44%	0.90
P-value: 1 year versus baseline	0.03	<0.001		0.007	<0.001	
P-value: 6 year versus 1 year	0.81	0.32		0.01	<0.001	
**Assistance with activities**						
Baseline	29%	11%	0.008	39%	6%	<0.001
1 year follow up	5%	3%	0.19	14%	9%	0.29
6 year follow up	12%	15%	0.55	11%	10%	0.86
P-value: 1 year versus baseline	<0.001	0.03		0.004	0.35	
P-value: 6 year versus 1 year	0.01	0.002		0.48	0.8	

#### Time spent on activities the previous day

**Figure 1:**
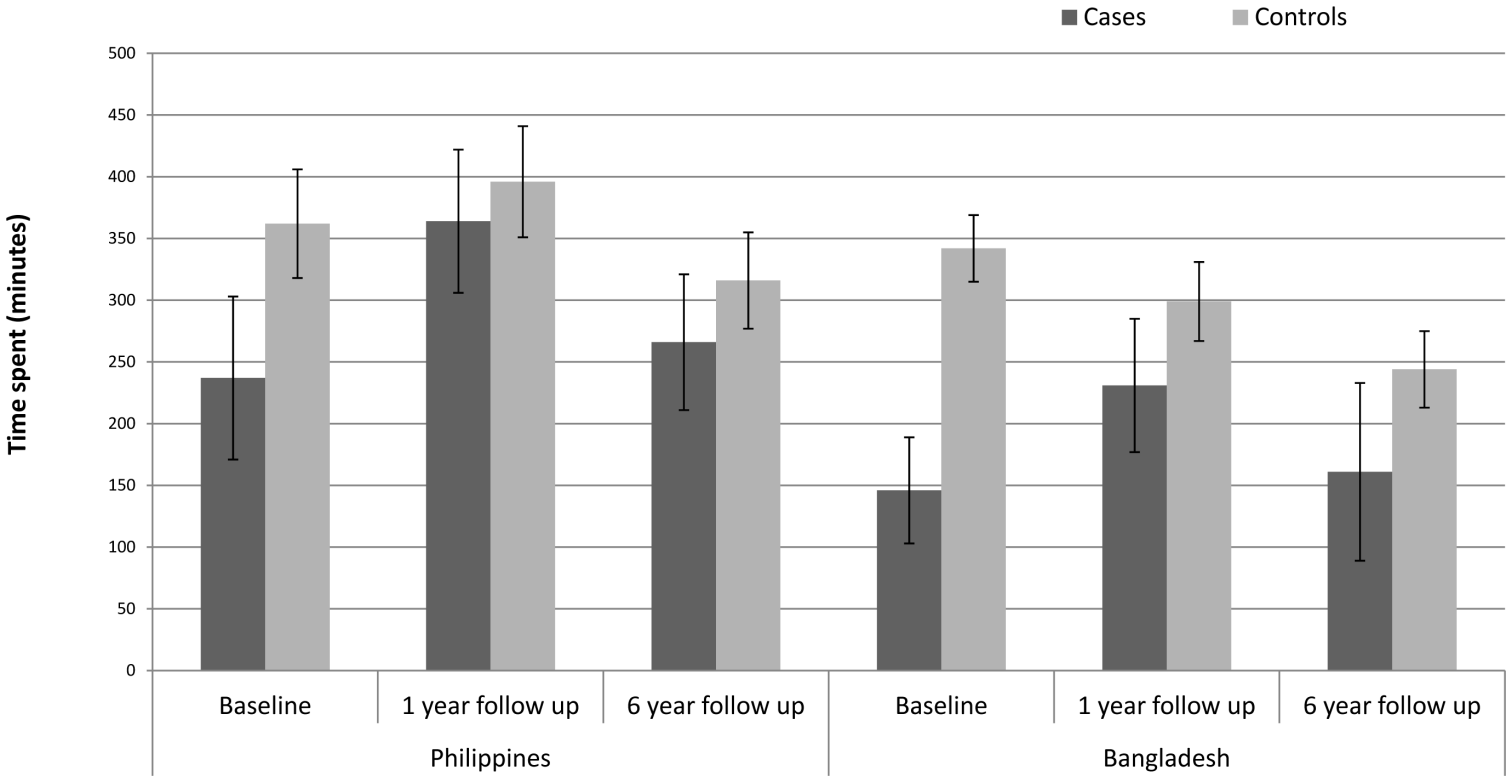
Time (minutes) spent on productive activities at baseline, one year and six year follow up among operated cases and controls.

At baseline cases spent significantly less time on productive activities (3 hours 57 minutes Philippines, 2 hours 26 minutes Bangladesh) compared to controls (6 hours 02 minutes Philippines, p = 0.002; 5 hours 42 minutes Bangladesh, p = <0.001, figure 1). One year after cataract surgery time spent on productive activities increased significantly among cases (to 6 hours 04 minutes in the Philippines, p = 0.003 and 3 hours 51 minutes in Bangladesh p = 0.004). However, at the six year follow up a significant reduction in productive activity time was observed among both cases and controls in both settings. In the Philippines there was no significant difference between cases and controls at either follow up, but in Bangladesh cases continued to spend significantly less time on productive activities than controls.

Time spent on leisure inside and outside of the house in both settings were similar for cases and controls at each time point (data not shown). In the Philippines, time spent in inactivity did not differ significantly between cases (3 hours 54 minutes) and controls (3 hours 13 minutes) at baseline (p = 0.55). Time in inactivity remained similar among cases at each time point, but increased among controls (to 4 hours 18 minutes at six years, p = 0.02). In Bangladesh, cases spent 2 hours more in inactivity than controls at baseline. At one year, this reduced significantly so that it was similar for cases (3 hours 8 minutes) and controls (2 hours 44 minutes, p = 0.25). However a significant increase was observed for both cases (to 4 hours 32 minutes, p = 0.006) and controls (3 hours 30 minutes, p = 0.007; case vs control p = 0.01) by the six year follow up.

#### Assistance with activities

At baseline cases compared to controls were two and a half times more likely to receive assistance undertaking activities in the Philippines (p<0.008) and six times more likely in Bangladesh (p<0.001, table 4). At both the one year and six year follow ups the proportion reporting assistance decreased significantly by at least a half so that there were no significant differences between cases and controls.

### Poverty

#### PCE

At baseline, cases had significantly lower PCE compared to controls (Philippines $22 vs $29 per person per month, p = 0.04; Bangladesh $15 vs $27, p = 0.02, table 5). One year after surgery PCE among cases increased compared to baseline to at least the level of the controls. This increase was sustained so that at the six year follow up PCE there were no statistically significant differences between cases and controls (Philippines $39 vs $37 p = 0.85, Bangladesh $23 vs $24 p = 0.85). In the Philippines there was a significant increase in expenditure between one and six years among both cases and controls (p<0.05).

**Table 5:** Per capita expenditure (PCE), assets and self-rated wealth at baseline and one and six year follow up for operated cases and controls.

	THE PHILIPPINES			BANGLADESH		
	Operated cases	Controls		Operated cases	Controls	
	Means (95% CI)	Means (95% CI)	P-value: Cases versus controls	Means (95% CI)	Means (95% CI)	P-value: Cases versus controls
**PCE (US$)**						
Baseline	21.73 (3.97–57.51)	29.01 (2.54–184.17)	0.04	14.95 (12.34–17.56)	26.90 (13.21–40.60)	0.02
1 year follow up	27.1 (5.37–134.39)	28.13 (3.27–108.14)	0.50	23.05 (16.55–29.56)	21.24 (19.42–23.07)	0.37
6 year follow up	38.9 (3.9–246.62)	37.4 (3.53–197.36)	0.85	23.29 (17.88–30.09)	24.29 (20.84–27.73)	0.85
P-value: 1 year versus baseline	0.14	0.96		0.001	0.02	
P-value: 6 year versus 1 year	0.008	0.004		0.52	0.74	
**ASSETS**						
Baseline	−0.25 (−0.97–0.46)	0.32 (−0.20–0.81)	0.20	−0.50 (−1.17–1.18)	0.33 (−0.16–0.82)	0.06
1 year follow up	−0.27 (−1.01–4.64)	0.27 (−0.23–0.77)	0.21	−0.52 (−1.20–0.15)	0.34 (−0.13–0.80)	0.05
6 year follow up	0.13 (−0.58–0.84)	0.15 (−0.37–0.66)	0.97	0.1 (−0.66–0.86)	0.14 (−0.36–0.64)	0.94
P-value: 1 year versus baseline	0.74	0.57		0.54	0.95	
P-value: 6 year versus 1 year	0.97	0.43		0.005	0.26	
**Self-rated wealth**						
Baseline	4.7 (4.3–5.0)	4.6 (4.3–5.0)	0.94	3.7 (3.1–4.2)	3.8 (3.3–4.3)	0.01
1 year follow up	4.5 (4.1–4.9)	5.1 (4.0–6.1)	0.46	3.8 (3.3–4.3)	4.4 (4.1–4.7)	0.06
6 year follow up	5.4 (3.2–7.7)	4.8 (4.4–5.3)	0.55	4.1 (3.5–4.6)	4.1 (3.8–4.4)	0.85
P-value: 1 year versus baseline	0.34	0.41		0.46	0.35	
P-value: 6 year versus 1 year	0.43	0.72		0.31	0.10	

#### Assets

At baseline, cases had lower asset scores compared to controls although this difference was only borderline significant in Bangladesh (−0.50 vs 0.33p = 0.06, table 5) and non-significant in the Philippines (−0.25 vs 0.32, p = 0.20, Table 2). Scores remained similar at the one year follow up. At the six year follow up, assets scores among cases had increased slightly so that they were similar to controls (Philippines 0.13 cases vs 0.15 controls, p = 0.96 Bangladesh 0.10 vs 0.14,p = 0.85).

#### Self-rated wealth

In Bangladesh at baseline, self-rated wealth was significantly lower among cases (3.7) compared to controls (4.5, p = 0.01) and this remained similar at the one year follow up (table 5). At the six year follow up, there was a small increase in scores among cases so that mean self-rated wealth was the same for cases and controls (4.1 for both, p = 0.86). In the Philippines there were no significant differences between cases and controls at any of the time points.

### Un-operated cases

Among cases who did not attend for surgery (‘un-operated cases’) the economic and activity variables either stayed similar or worsened over the two follow ups (data not presented). For vision related Qol there were some small increases in scores for un-operated cases, although these were considerably smaller than for the operated cases. The proportion reporting problems with EQ-5D scores remained similar over time, with the exception of mobility and activities in Bangladesh in which there were some reductions in reported problems.

## Discussion

The cataract impact study explored the effect of cataract surgery on three different but interlinked outcomes: health related quality of life, daily activities and economic poverty, in Bangladesh and the Philippines. Prior to surgery people with cataract visual impairment had poorer vision and health related QoL, were less likely to undertake and spent less time on productive activities, were more likely to receive assistance with activities and were economically poorer compared to controls without vision impairment. One year after surgery there were improvements in each of these outcomes so that the operated cases were similar to controls. However, it was unclear whether these benefits would be sustained in the long term, particularly considering the older age group of the study population who may be more likely to experience other co-morbidities. The current study has indicated that the increases in vision and health related QoL, participation in productive activities and PCE and reduction in reported assistance with daily activities were still evident after six years. Average daily time spent on productive activities decreased among both cases and controls.

We measured three different indicators of poverty: PCE, asset ownership and self-rated wealth. One year after cataract surgery increases in PCE were observed among operated cases, but asset ownership did not change. However, after six years there were some increases in asset scores in both settings among operated cases. This finding is intuitive as asset ownership is a longer term measure of poverty than expenditure and therefore more time is needed for households to accumulate possessions. Improvements in self-rated wealth were not observed. This may be because as it is a measure of perceived wealth relative to other households in the community which might be harder to change or because there was a limited variation in this variable so that we were under-powered to detect a change.

The activity data collected in this study provide possible explanatory routes for the observed improvements in PCE and assets. One route is through the increased productivity of the individual operated for surgery: six years after surgery a higher proportion of cases remained engaged in productive activities than at baseline. Productive activities included paid work and also non-paid activities which can make significant economic contributions [24]. There were some reductions in participation in and time spent on productive activities among both cases and controls between one and six years. This is not surprising in a population of older adults (approximately half of participants were aged 70 years and above in both settings) where health may be deteriorating over time. A second possible route is through increased productivity of other household members as a result of increased independence of the operated case. This is supported by the sustained reduction in reported assistance from others with daily activities six years after surgery. Further research on the impact of cataract surgery on the daily activities of other household members is needed to confirm this.

There are a limited number of longitudinal studies with which to compare these findings. Our findings support two studies in India which showed increased household income [25] (Finger, 2012) and engagement in income generating [25], [26] and household activities [26] within a year following surgery. Our current study provides additional evidence that this positive impact is sustained in the longer term.

We also found evidence of substantial sustained benefits of cataract surgery on vision and generic health related quality of life. There were some small reductions in quality of life measures between the one and six year follow up among cases and controls, however this is unsurprising for this age group where deteriorations in vision and health may be expected. The improvements in vision related QoL one year after surgery are consistent with two studies in India [25], [27]. Previous studies on generic HRQoL are more mixed, with some studies in high-income settings finding evidence of improvement [28], [29] and others showing no change following cataract surgery [15], [30], [31]. Few studies of the long term impact could be identified. However, our study supports findings by Lundqvist et al that visual function scores remained stable 10 years after cataract surgery [14]. A study in Australia found long term improvements in mental but not physical domains of the SF-36 health related quality of life instrument [15].

There were a number of limitations to this study. Uptake of surgery was lower than expected and loss to follow up was around 50% at the six year follow up. The sample size was therefore small which has implications for the tests of statistical significance (of which there were many) as the study may have had insufficient power to detect statistically significant true differences in the outcome variables. There were few differences in socio-demographic characteristics between operated and unoperated cases and those included/lost to follow up reducing possible impact on external validity. The exception was that the participants who did not attend for surgery (both settings) and cases who were lost to follow up (Bangladesh only) were slightly older. We therefore cannot rule out the possibility that if more of the unoperated cases had attended surgery and if fewer participants were lost to follow up the increases in PCE and productive activities might have been smaller.

There were some improvements in vision and generic HRQoL among unoperated cases over time although this was considerably smaller than for operated cases. The reason for this is unclear, but may reflect adaptation to their level of vision loss over time or that having decided against surgery they under reported difficulties with visual functioning at the follow up to discourage survey team members from trying to suggest they should attend.

Finally there were potential limitations with the activities data in terms of the reliance on recall and the collection of data for a single day which may not represent a typical day. Further although data were collected during the same climatic season at the three time points, they were not always collected during the same month and time of year, which may influence time-use patterns.

Strengths of this study are that it was conducted in two different countries and used standardised data collection tools at three different time points. Study participants were population based rather than from hospitals which increases the generalisability of the findings. We included matched controls without vision impairment and in general their outcomes remained relatively stable or worsened over time. This provides support to the changes among operated cases being due to cataract surgery and not to other events.

### Conclusion

The six year follow up of the Cataract Impact Study is the first study to explore the long term impacts of cataract surgery on people's lives in low income settings. This study has indicated that there are sustained benefits of cataract surgery to a population of older adults in low-income countries in terms of health related quality of life, daily activities and household economics and highlights the importance of cataract surgery in improving the lives of people in the long term.
